# Authenticity and presence: defining perceived quality in VR experiences

**DOI:** 10.3389/fpsyg.2024.1291650

**Published:** 2024-04-19

**Authors:** Asim Hameed, Andrew Perkis

**Affiliations:** Department of Electronic Systems, Norwegian University of Science and Technology (NTNU), Trondheim, Norway

**Keywords:** virtual reality (VR), user experience, user-perceived quality, presence, plausibility, believability, authenticity

## Abstract

This work expands the existing understanding of quality assessments of VR experiences. Historically, VR quality has focused on presence and immersion, but current discourse emphasizes plausibility and believability as critical for lifelike, credible VR. However, the two concepts are often conflated, leading to confusion. This paper proposes viewing them as subsets of authenticity and presents a structured hierarchy delineating their differences and connections. Additionally, coherence and congruence are presented as complementary quality functions that integrate internal and external logic. The paper considers quality formation in the experience of authenticity inside VR emphasizing that distinguishing authenticity in terms of precise quality features are essential for accurate assessments. Evaluating quality requires a holistic approach across perceptual, cognitive, and emotional factors. This model provides theoretical grounding for assessing the quality of VR experiences.

## 1 Introduction

Virtual reality (VR) is historically preoccupied with delivering realistic and immersive experiences, seamlessly transporting us into immersive worlds that blur the lines between the real and the virtual along the virtuality continuum (Milgram et al., 1995). It belongs to a range of emerging technologies that generate omnidirectional extended reality (XR) experiences for users. These are either mixed reality (MR) technologies that overlay digital images and information on the physical context or create a new reality by completely occluding the natural context, like VR (LaValle, 2016). In the past, the focus has remained on the perceived quality of VR, namely *presence* and *immersion* (Lombard and Ditton, 1997; Nilsson et al., 2016). Recently, we see the discourse expand that scope to include *plausibility* and *believability* as crucial judged quality aspects of VR experiences (Slater, 2018; Weber et al., 2021). These terms describe virtual environments in their lifelikeness, whose behavior makes sense and allows one to suspend disbelief. Both terms are interchangeably used to comment on the credibility of a VR experience in the degree to which a VR environment adheres to rules, constraints, and logic that harmonize with what users expect (Skarbez, 2016). Similarly, other terminologies, such as “coherent” and “congruent” also come up to describe the predictability and consistency of features and behaviors within virtual worlds. This work focuses on users' subjective judgments of a VR experience's credibility—referred to as its *authenticity*. We recognize that assessing the experiential quality of VR is not a one-dimensional task and concepts like plausibility and believability require invested research. It is also important to highlight that these concepts must not be reduced to singular notions of coherence or realism alone. At the same time, we believe that the use of both terms, though critical, often blurs at the edges, giving rise to conceptual confusion. Within this paradigm, we ask: how can the concepts of plausibility and believability be clearly defined and differentiated within the broader notion of authenticity in VR experiences? Further, what roles do coherence and congruence play in complementing plausibility and believability to evaluate the overall quality of VR experiences? To this end, the paper will first separate the two terms and accurately outline their differences and connections. Secondly, we propose a structured hierarchy that defines plausibility and believability as subsets of the overarching concept—authenticity. Through this, we hope to delineate the boundaries and intersections of these terms. Finally, we introduce coherence and congruence as quality functions complementing plausibility and believability. This dynamic interplay underscores the importance of considering both the internal and external logic of a VR experience and the alignment of its stimulus to the users' perceptions and experiences in evaluating its overall quality. The proposed authenticity paradigm integrates previous frameworks on presence, realism, and plausibility. We synthesize these perspectives into a cohesive structure that can guide the analysis and design of high-quality VR experiences. Adopting a nuanced perspective that approaches authenticity and presence as experienced quality can enhance theoretical clarity and provide stronger empirical grounding for studying user experiences in VR.

## 2 VR—Realistic, plausible, and believable

When assessing the overall quality of VR experiences, we have had a historical preoccupation with *realness* or *realism*. Realism in VR expands from the fidelity of available stimuli to the perception of how closely a virtual environment (VE) imitates the real world (Alexander et al., 2005; Lombard et al., 2009). VR experiences are commonly assessed in terms of two crucial dimensions: presence and immersion (Schuemie et al., 2001; Biocca, 2002; McMahan, 2003; Lombard et al., 2009; Slater, 2018). The richness of the VE profoundly influences both of these facets—its visual, aural, and fidelity—which play a pivotal role in captivating users and enhancing their sense of immersion (Steuer, 1992). Engaging a user with rich and exclusive sensory stimulation inside a head-mounted display (HMD) achieves a sense of *presence*—an objective property of the system (Bowman et al., 2012)—associated with a vivid sense of being “there” in the virtual world, interacting with virtual objects, engaging with virtual characters, and feeling emotions within the simulated world. The prevailing discourse in VR has often leaned heavily on the prominence of presence (Kim and Biocca, 1997; Lee, 2004) as the primary construct of a subjective experience of feeling transported into a virtual world. It is a psychological state influenced by the user's expectations, beliefs, and experiences. Immersion (Witmer and Singer, 1998; McMahan, 2003) meanwhile are the technological (or system) aspects that surround the user, as mentioned before. It is the extent to which any user would feel absorbed in the virtual world owing to its ability to produce and render scenarios and experiences with a high degree of realism (visual and audio fidelity), responsiveness (interactive fidelity), embodiment (sensorimotor stimulation and feedback) (Steuer, 1992; Baños et al., 2004; Kilteni et al., 2012). Presence has long been considered the defining quale of VR; however, an overemphasis risks overlooking other critical elements of the overall user experience. As VR technology advances and its applications expand, it becomes increasingly evident that presence alone is an insufficient framework to capture the richness and complexity of VR experiences fully.

Multiple other works exploring complementary phenomena influencing VR experiences share this point of view. Earlier on, Slater (2009) conceptualized a theoretical framework with two orthogonal components, namely *place illusions* and *plausibility illusion*. Place illusion denoted presence, while the additional plausibility illusion referred to the realism and likelihood of a VR scene. In their terms, “the overall credibility of the scenario being depicted” juxtaposed with user expectations, delivering an impression that the system-generated events were occurring. Later iterations of the concept have used both the term plausibility (Rovira et al., 2009; Hofer et al., 2020) as well as other classifications for the same theoretical principle; reality judgment (Baños et al., 2000), perceived realism (Lombard and Ditton, 1997; Schubert et al., 2001), coherence (Skarbez et al., 2017), and authenticity (Gilbert, 2016). These works view plausibility as a higher-order cognitive operation that involves a judgment on the credibility or authenticity of the VR scene, which is reflected by its consistency and the extent to which it meets a user's expectations.

Looking in detail at plausibility is essential to differentiate between various quality aspects of the phenomenon. For Skarbez (2016), this translates to when a VE projects situations that appear apparent to the users based on their existing knowledge of the world. Such knowledge can include their understanding of both the real world and their knowledge of the fictional world depicted inside VR. Internal plausibility is how well it follows its rules and makes sense within its framework. External plausibility is how consistent it is with real-world knowledge and whether it matches a user's understanding of the real world (Busselle and Bilandzic, 2008; Hofer et al., 2020). An updated review, published by Slater et al. (2022), added depth to their initial conceptualization by specifying different instances of plausibility inside VR: a reactive environment that responds to actions, contingent interactions that happen in relation to the user, and coherence with users' expectations based on their experiences and knowledge. A more recent contribution by Latoschik and Wienrich (2022) looks at plausibility alongside congruence—how we feel about the experience and how well it matches our expectations. Their model considers congruence as the objective match between the information processed by the user and their expectations at the sensory, perceptual, and cognitive levels. Plausibility results from the evaluation of congruence across the three levels. Sensory congruence is how well the experience matches our senses. Perceptual congruence is how well the experience fits our understanding of how the world functions. Finally, cognitive congruence is how well the experience matches our beliefs and expectations. Weber et al. (2021) have identified plausibility under the concept of *perceived realism*, which extends to (1) the realism of objects, sounds, and scenes in terms of their congruence to real-world textures, proportions, details, etc. (2) the plausibility of story and characters, evaluating their consistency rather than factual accuracy, and (3) judgment about the naturalness of interactions.

Another term often interchangeably used with plausibility is that of *believability*. Closely related to the historic literary notion of the “suspension of disbelief” on the part of the audience/reader to suspend their judgment concerning the implausibility of a given narrative (Chandler and Munday, 2011). The idea that audiences are willing to accept the premises of a fictional work, even if they are fantastical or unrealistic, as long as that world and its characters feel subjectively accurate and coherent enough. For VR, the suspension of disbelief is essential for creating believable experiences. If the virtual world is believable enough, users will accept its artificiality and immerse themselves in it, just as a reader would in a fictional story. Sheridan referred to it as “the active imagination in suppressing disbelief (and thus enhanced believability)” (Sheridan, 2000). Believability is also defined as elements operating at various levels of realism—sensory, perceptual, and emotional—manifested through realistic visual and aural effects, a consistent VE that allows natural interactions, as well as aesthetic, dramaturgical, and emotional aspects of the VR experience (Magnenat-Thalmann et al., 2005; Papagiannakis et al., 2005; Bogdanovych et al., 2015).

We recognize the significant contribution of the frameworks and models described in the previous section. Concurrently, we recognize the necessity of consistently refining concepts to enhance clarity, especially because using broad and repetitive terminologies adds uncertainty to quality assessments. We believe maintaining distinct terms for plausibility and believability is necessary to explain fully the characteristics and influences shaping VR experiences. This distinction also aligns with semiotic principles, given that the elements outlined in the frameworks and models discussed previously correspond to separate semantic and syntactic categories (Barricelli et al., 2016). Therefore, an explication using precise language for describing quality aspects and avoiding confusion between key constructs and factors is important. Further, we agree with the contention that fixating solely on presence does not encapsulate the multifaceted nature of VR experiences (Gilbert, 2016). In the following section, we propose a recalibration of focus toward plausible and believable VR experiences that we present as subsets of a quality model for *authenticity*.

### 2.1 The Presence–Authenticity Dyad

Gilbert (2016) described authenticity as how well the VR environment mirrors the expected regularities of the world it is trying to represent (Bowman et al., 2012). How faithfully does it replicate the behaviors, relationships, and rules consistent with its purported context? How closely an entity aligns with an individual's expectations, cognitive schemas, prior knowledge, personal experiences, preferences, and interaction reciprocity (Bucolo, 2004; Weibel et al., 2010). The longer a user stays in a VR environment, the more likely their initial sense of wonder will give way to a heightened awareness of the environment's authenticity. Once familiarized, users begin to notice incongruities in the VR setting. For instance, the inability to physically interact with virtual objects in an intuitive way (Hameed et al., 2021), the failure of non-player characters to respond to the user's existence (Rovira et al., 2009), or a disjunction between the realism of the user's avatar and the aesthetic of the world they inhabit (Slater, 2017). This shift from an initial enchantment to a heightened critical awareness of its features reflects the various quality aspects that influence the assessment of a VR experience. It highlights that while a robust place illusion is necessary, it may prove shallow and lose its spell if the virtual world gives the impression of being inauthentic.

Considering this, we introduce the “Presence-Authenticity Dyad,” recognizing authenticity as a complementary dimension to presence and crucial for evaluating the quality of VR experiences. In similar vocabulary to that which characterizes presence as a feeling of “being there,” this work defines authenticity as “*a sense of ‘trueness and genuineness”'* felt in a virtual place.

In agreement with Lee (2020), we see it as users' individual judgment on the virtual world's trueness and genuineness regarding its stimuli, content, and behavior. We expand this to include two subtypes of believability and plausibility. Despite their interchangeable use, they refer to related but distinct characteristics in the virtual. Both contribute to overall authenticity and presence. We define them as follows:

- *Plausibility* is the extent to which a VR experience can be logically explained and remains consistent with real-world principles. What's happening is real. It refers to the degree to which the VR environment and its contents exhibit logical congruence and follow common sense. For example, perceptual constancy, the consistency of its physics, etc. Plausibility operates at the syntactic level, reflects in logical consistency, and has more objective thresholds. It reflects the trueness of the depicted world. An experience is plausible if the environment and its contents remain rational and conform to the principles of its rules-based reality.
- *Believability* is how much a VR can deliver an experience with the realism and internal coherence required to make it feel believable for the user. It goes beyond mere visual fidelity and taps into the user's emotions, senses, and overall engagement with the virtual world. If it is convincing, it's happening. Factors include narrative logic, engaging gameplay, etc. Believability carries semantic elements, includes emotional resonance, and aligns variably based on the subjective perceptions of the user. It reflects the genuineness of the depicted world in its subtle details and nuances that mimic reality and support a “suspension of disbelief,” even if the experience itself is fantastical or fictional.

### 2.2 A quality interpretation of authenticity

**Figure 1:**
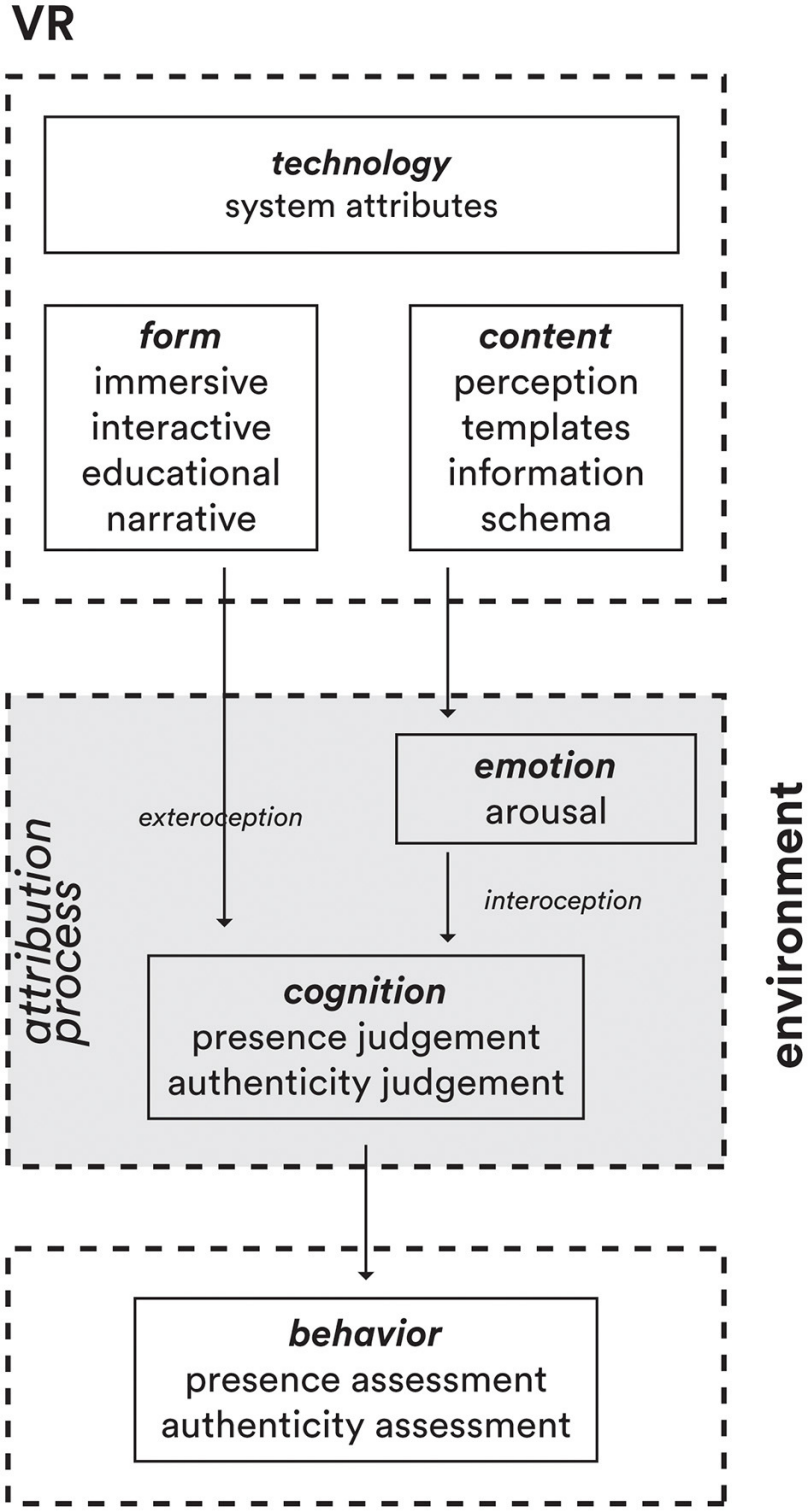
The attribution process leading to judgment inside VR, adapted from Diemer et al. (2015).

In line with the notion that presence constitutes a subjective sensation contingent upon the immersive attributes of a system, it becomes evident that a system must first facilitate immersion to establish the semblance of “being there.” In a parallel vein, one can argue that authenticity reinforces the illusion engendered by the VR system, thereby influencing the efficacy of a VR experience. Presence and authenticity are two distinct facets of a VR experience, with multiple quality aspects underpinning the two phenomena (see Figure 1).

#### 2.2.1 On quality formation

Quality involves perceptual, cognitive, emotional, and evaluative processes determining one's conscious perception of things. The word stems from qualia—qualitative, phenomenal aspects of consciousness and subjective experience (Gregory, 1996)—in the form of sensations, feelings, and mental imagery that reflect what it is like to experience something. They may include impressions of goodness, beauty, desirability, and virtue that arise in consciousness when encountering something (Shoemaker, 1990). Jekosch (2005) refers to *experienced quality* as the result of a mental evaluation where someone compares the actual composition of something to their expected or ideal composition. In other words, experienced quality is the subjective judgment of how well an entity's perceived composition aligns with desired expectations (Blauert and Jekosch, 2012). The term “entity” denotes any object or event, material or immaterial, that becomes an object of perception.

This may be straightforward in the physical realm where entities have objective physical attributes (or *quality elements*) that can be measured, e.g., display resolution. But in the subjective realm of perception, entities exhibit psychological features (or *quality features*) such as vividness and richness. A subject (user) perceives an entity's quality features and compares these to their internal ideals and expectations, which shapes a conscious impression of the entity's overall quality (Uhrig, 2021). Möller (2023) categorizes quality elements and features into complementary factors and aspects. For example, technical factors like throughput and jitter can affect perceptual aspects like immersion and embodiment.

Jekosch (2005) reformulations further identify a fundamental distinction between two facets of quality perception: *perceived quality* and *judged quality* (Uhrig, 2021). *Perceived quality*—akin to low-level thinking—is an immediate impression formed upon encountering a stimulus. Damasio (1995) argue that such a swift quality assessment upon encountering a natural environment or a technological stimulus need not require deep cognitive processing but results from integrating basic perceptual features into an abstract evaluation. For example, a user may perceive the quality of a depicted scene as poor upon first seeing it but without consciously analyzing why. Despite being an initial reflexive impression, perceived quality can still be intentionally contemplated and judged after the fact. This evaluative process produces a quality judgment that reflects cognitive analysis. The subjective experience of this quality judgment is termed the *judged quality*. Since cognitive evaluation activates complex associations and interpretations, judged quality encompasses richer perceptual, conceptual, and affective content than perceived quality. It is influenced by conscious analysis and reflection, not just direct perception.

**Figure 2:**
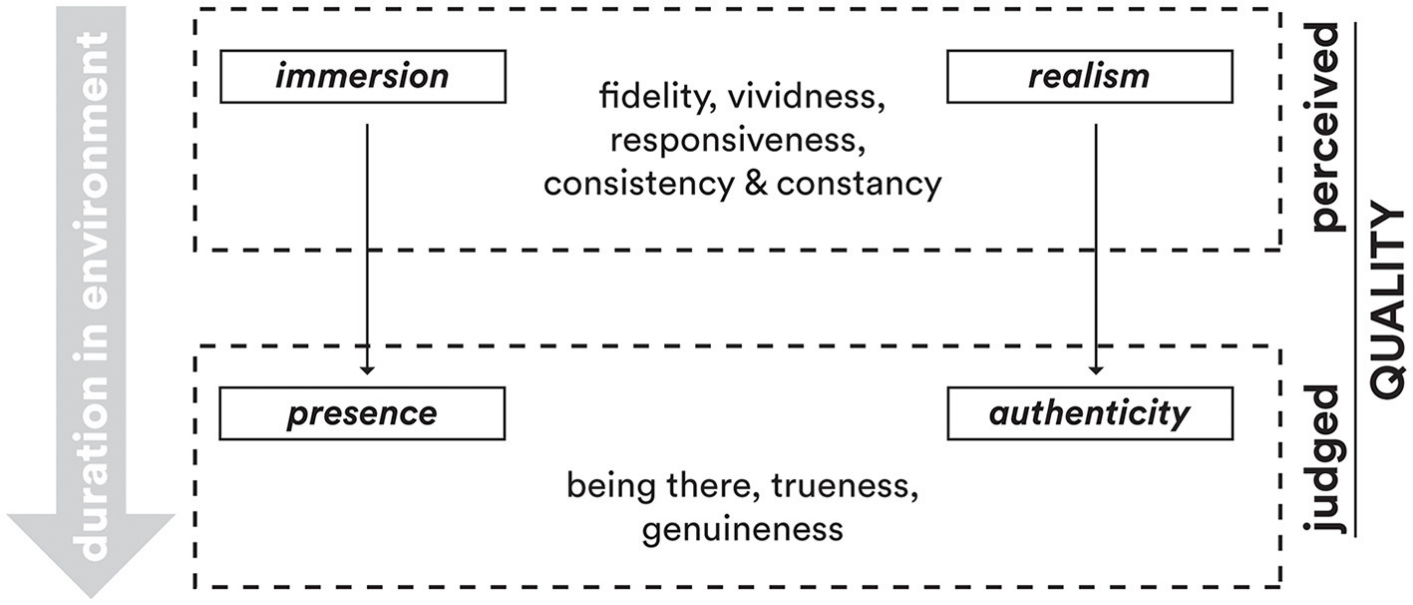
Judgment process from perceived quality to judged quality. Immersion-related attributes give rise to a sense of presence, whereas perceived attributes of consistency and constancy (or realism) foster an authenticity about the experience.

The distinction between perceived quality and judged quality relates to the notion of experience, which can be defined as “the stream of perceptions (of feelings, sensory percepts, and concepts)” that occur in a given situation (Möller and Raake, 2014). In this respect, perceived quality aligns with the immediate experience of impressions and sensations within the VR world. It is an intuitive and phenomenological part of experiencing that world. However, judged quality goes beyond just experiencing. It requires additional cognitive processing to evaluate and consciously judge the quality of the depicted VR world (see Figure 2). So, while perceived quality is embedded in the direct experience, judged quality emerges from reflective analysis and interpretation of the VR experience. The former is instant, while the latter involves extra mental effort to reach an overall quality assessment.

#### 2.2.2 Quality aspects of authenticity

Earlier in this paper, we defined authenticity as the trueness and genuineness of the displayed VR place. As evident, both words imply a deliberate judgment of the depicted place on the user's part. This is unlike a user's more immediate, intuitive impression of the VR setting's various sensory inputs and atmospherics. In fact, upon first encountering, users will be relying on sensory percepts to discern the visuals, sounds, and vectors available in the VR space. However, initial quality perceptions simultaneously evolve as they are compared to quality features internally desired by the user (expectations). We argue here that even at the most nascent stages of their embodied encounter, the *experienced quality* of the virtual world is enough to imbue a feeling of presence (ephemeral as it may be). The longer the visitors stay in the immersive VR world from here on, the more their awareness is heightened with respect to the lifelikeness, interactive intuitiveness, and audio-visual synchronicity, etc. of the VR setting. Quality judgments on trueness and genuineness entail conscious assessments of the virtual world's congruence and coherence and reflect this heightened state of intentional and reflective cognitive processing. Both quality descriptions go beyond initial impressions to include complex and nuanced evaluations of whether the VE maintains its integrity, i.e., credibility. These judgments pertain to perceptions and desires but also carry emotional and evaluative dimensions.

Trueness is defined as “conformity to reality and actuality” or “agreement to fact and reality” (Webster, 2014). It is focused on the accuracy of the information following reality and reflective of facts. Conversely, genuineness is defined as “the quality of being honest and sincere” and “the quality of being real and exactly what it appears to be” (Webster, 2014). It goes beyond mere accuracy and delves into sincerity. It encompasses the quality of being real and without pretense. In terms of determining experiential quality in VR, authenticity must then be understood as the sum of the factual accuracy of the world as well as the sincerity of its self-expression. Its trueness is evidence-based (objective), whereas its genuineness is internally driven (subjective).

In terms of a VR experience, both trueness and genuineness are distinct quality features of the authenticity of that experience. As such, we associate them with the quality aspects that determine authenticity, i.e., plausibility and believability, respectively. Trueness speaks to the plausibility of a VR experience and genuineness reflects its believability. Table 1 charts the differentiation of quality goals for the two aspects, the factors influencing them, and some evaluation methods to assess them. Moreover, we refer to the terms *congruence* and *coherence* as functions of the two quality aspects that specify either the fulfillment or nonfulfillment of authenticity. We ascribe the term coherence to believability and congruence to plausibility. The former describes an inner connectedness or integration of meaning within something, while the latter refers to an alignment or matching between two or more things (virtual-to-real). Our appropriation of both terms is consistent with how they regularly appear in VR research. Most definitions of coherence relate to Skarbez et al. (2017), who have referred to it as the internal consistency of a virtual experience and defined it “as the set of reasonable circumstances that the scenario can demonstrate without introducing unreasonable circumstances.” How well the parts of something fit together logically. A coherent experience should reflect consistency through its story, visuals, sounds, characters, tasks, etc. Its parts must understandably indicate a unified whole, with ideas that make sense together. Correspondingly, the term congruence has been borrowed from environmental psychology to depict an agreement or consistency between things and is defined as “the degree to which different cues fit with each other” or “a similarity between perceptual variables” (Maffei et al., 2016; Flavián et al., 2021). Congruence may carry the processing of physical and relational information reflected in matching the logic, physical behaviors, and limitations within a virtual experience.

**Table 1:** Key objective and subjective determinant for evaluating *plausibility* and *believability* in VR.

	***Plausibility***	***Believability***
*Definition*	Adheres to real-world principles, feels rational and explainable; trueness	Resonates with perceptions and emotions, feels subjectively “real”; genuineness
*Function*	Syntactic; Logical congruence	Semantic; Internal coherence
*Descriptives*	Perceptual constancy of objects Physics consistency Logical cause-and-effect chains Multi-sensory alignment Visual grounding Situated acoustics Fast interactive responsiveness	Scenario logic Atmospherics and randomness Narrative and Stylistic cohesion Environmental imperfections Nuanced reactions Resonance with memories and emotions Subjective presence
*Factors*	Stable geometry and optimized models Unrealistic forces and behavior Penetrations and incorrect scaling Audio synchronization and acoustics Lighting/shadow matches source Assets situated logically	Physically based rendering PBR materials and textures Detailed assets/expressive characters Subtle environmental cues Realistic audio sampling Natural conversational flow
*Evaluations*	Quality Metrics to evaluate 3D models Metrics for physics simulations Test logical contradictions Examine against physical rules Check sensory alignments Detect affordance mismatches	User testing and feedback Track user behavior Monitor user performance Assess emotional responses Review ecological realism Survey narrative realism and disbelief

**Figure 3:**
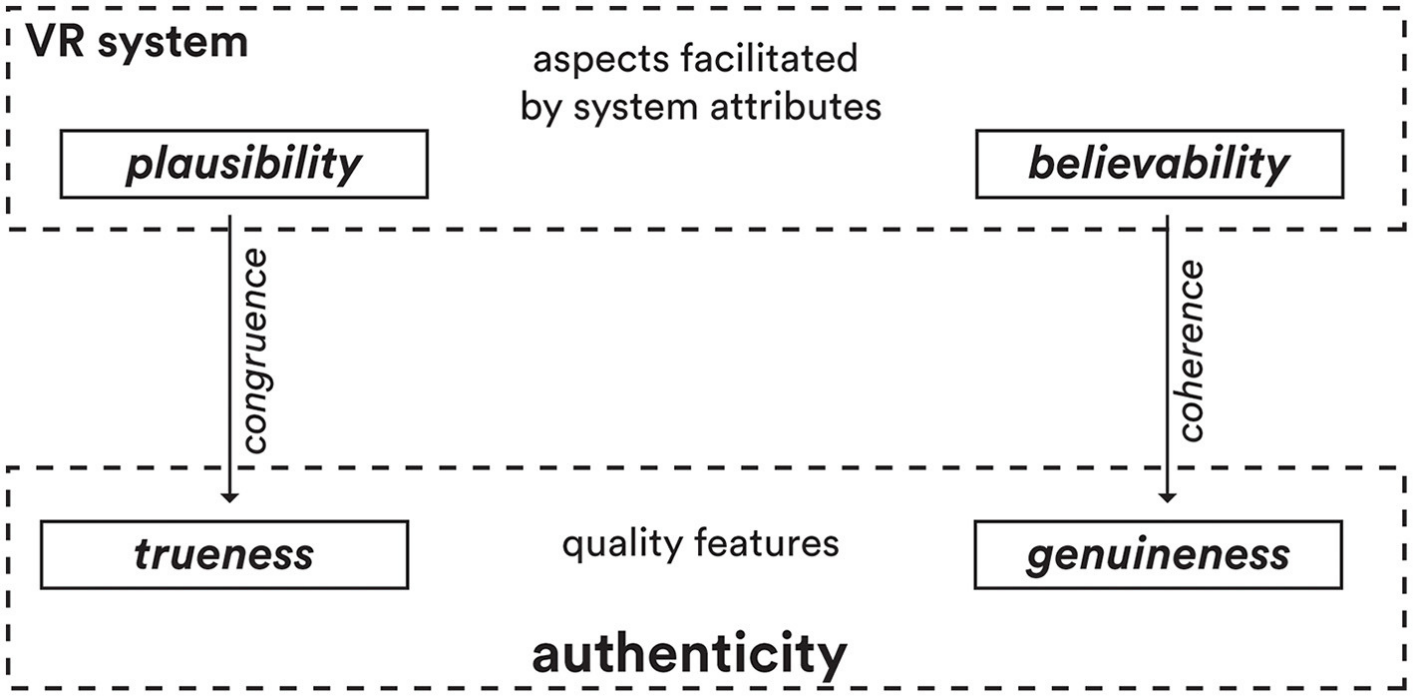
Quality formation of experienced *authenticity* inside VR.

Our revised understanding of authenticity in VR suggests that while a user's initial engagement may stem from a feeling of presence within a computer-generated environment, a lasting impact of the VR experience hinges on its ability to instill a sense of genuineness and trueness within the virtual world (see Figure 3). It must carry qualities that inspire belief in its meaning and truth to sustain immersion. More than its illusions, a VR experience must resonate in its essence and significance to the user.

## 3 Discussion

In this paper, we explored the multifaceted nature of quality assessment for immersive VR experiences by drawing attention to a conceptual distinction between perceived quality and judged quality. We proposed identifying authenticity as a key dimension of quality perception complementary to the feeling of presence. Existing literature on quality assessments of VR experiences emphasizing presence has often overlooked authenticity. This has led to multiple conceptualizations and questionnaires that remain preoccupied with system factors facilitating immersion and generating a one-time sense of “being there.” There is a need to explicitly differentiate between realism as the fidelity and richness of the mediated environment vs. authenticity as the trueness and genuineness of virtual worlds. Clearly distinguishing these as two quality facets will allow for more precise definitions and measurement instruments.

To this end, we distinguished plausibility (trueness) and believability (genuineness) as distinct yet complementary aspects contributing to a VR experience's overall authenticity. Plausibility refers to the objective, logical congruence of the virtual world in adhering to real-world principles, natural laws, and common sense rationality. It operates at a syntactic level, reflected in consistencies like perceptual constancy of objects, accurate physics simulations, and logical cause-and-effect chains unfolding within the environment. In contrast, believability is more subjective, relating to how genuinely “real” the experience feels to an individual user based on their personal perceptions, prior experiences, and evoked emotions. While plausibility entails maintaining objective rules and realism, believability hinges on semantic details, stylistic nuances, and resonant engagement that suspends disbelief and facilitates immersive psychological involvement, even if the content is fantastical or imaginative. Thus, plausibility cues are more binary while believability varies across users.

It is important to highlight that VR experiences need not always mirror real-life scenarios. Experiences could involve unrealistic, fictional, or imaginative elements. Yet if these elements interact with the user congruently and coherently, they can feel authentic. For example, a virtual world that simulates real-life settings must meticulously adhere to real-world nuances and principles. In such a context, the VE should respect the laws of gravity, ensuring that objects behave as they would in the physical world. Conversely, objects may defy gravity in VR to provide a fantastical experience in a zero-gravity environment. Since such unnatural defiance aligns with the intended narrative, it will be acceptable in that depicted world. These flights of imaginative engagement encourage a willful “suspension of disbelief,” which may be construed as a momentary recalibration of one's preconceived notions. Within this framework, individuals can momentarily adopt cognitive predispositions that harmonize with the fictitious realms they are immersing themselves in. This cognitive adaptability allows users to traverse and comprehend various VR experiences, from the meticulously realistic to the purely fantastical. This helps them appreciate the diversity of content and modalities within VR. Users bring their prior beliefs, but once inside the VE, new sensory input is integrated with these priors to update their beliefs, which influences their perception of the environment's realism (Triantafyllou et al., 2014; Gilbert, 2016). For example, a user entering a virtual forest will compare the sensory input (like the appearance and sounds) with the priors of a real forest. If it aligns, the virtual forest will maintain its authenticity. The need for authenticity in VR extends to the consistency of interactions, relationships, and elements within the virtual space. If users perceive inconsistencies, mismatches in coherence, or behaviors that contradict their expectations, their sense of authenticity can be disrupted (Biocca and Delaney, 1995). This could, in effect, lead to a break-in-presence or a decrease in the overall quality of the VR experience.

Evaluating quality necessitates a holistic approach spanning perceptual, cognitive, and emotional factors. As users spend more time immersed in a virtual environment, perceived quality gives way to judged quality as inconsistencies become apparent. Achieving high-quality VR experiences involves optimizing both low-level processing and higher-level functions of congruency and coherence assessing events and interactions within the VE. Adopting a nuanced perspective that approaches authenticity and presence as experienced quality can enhance theoretical clarity and provide stronger empirical grounding for studying user experiences in VR. Below, we extend this discussion to briefly describe various technical and human-centric factors influencing plausibility and believability.

**Evaluating plausibility**

The technical factors contributing to a positive and immersive experience in VR are perceptual constancy, aliasing and sampling, audio synchronization, and physics consistency. Perceptual constancy ensures that objects maintain their appearance despite changes in environmental conditions (Coren et al., 2004; Jerald, 2015) while aliasing and sampling reduce visual artifacts like jagged edges and pixelated textures (Gibson and Mirtich, 1997; Lessiter et al., 2001). Audio synchronization improves the authenticity of the aural experience (Guastavino et al., 2007), while physics consistency requires emulating real-world scenarios with physics engines that behave realistically (Hummel et al., 2012). One suggestive evaluation approach uses quality metrics to assess 3D models and physics simulations based on their real-world physical properties and material types. Another recommendation is to evaluate how well the system adheres to established rules and cause-and-effect relationships within the defined world logic. This evaluation should be examined against physical rules and check sensory alignments (Chen et al., 2019). Additionally, it is suggested to use metrics such as collision detection, object interactions, and gravity behavior to assess the accuracy and realism of physics simulations in the virtual environment (Jiang et al., 2018). Lastly, tracking object interaction frequency and accuracy can help identify instances of affordance mistakes and analyze control mechanics to improve user experience (Hameed et al., 2021). Subjective measures involve questionnaires that assess how realistically users perceive the virtual world and how well it aligns with their prior expectations of similar environments (Regia-Corte et al., 2013). Self-reported measures can be employed to investigate emotional responses to implausible or nonsensical events. The overall pleasantness and engagement of the virtual experience can be assessed through questionnaires and surveys, gathering user feedback on their positive and negative affective responses to the features and elements within the VR environment (Möller et al., 2013; Hameed et al., 2023).

**Evaluating believability**

One of the crucial aspects of believability remains the use of high-fidelity stimuli, which includes various features such as render quality, physics engine, and spatial audio (Skarbez, 2016; Slater et al., 2022). In addition, internal coherence and consistency are essential, which means that all elements within the virtual world should make sense and be consistent with the established setting and rules (Lepecq et al., 2009). Details that reflect real-world experiences, such as environmental imperfections and character animations, can significantly enhance the feeling of naturalness within the environment (Loomis, 2016). Moreover, the complexity and realism of scripted events or narratives in the virtual world should remain logically consistent, and users should anticipate what comes next (Llobera et al., 2013; Skarbez et al., 2020). The sense that actions and experiences within the virtual world have value or significance also adds to their meaningfulness, which can heighten users' cognitive absorption and emotional engagement (Murray et al., 2007; Beckhaus and Lindeman, 2011). To evaluate virtual assets, animations, and environments, use industry benchmarks and standards (Otto et al., 2019). Measure the world's size, complexity, and dynamism with metrics like the number of environments, objects, and paths (Lugrin et al., 2013). Assess the level of detail and use of sound effects to enhance the virtual world's believability (Tran et al., 2021). A human-centric factor that contributes to the believability of a virtual world is the suspension of disbelief, which refers to users' willingness to temporarily accept the virtual world as real even though they know it's not (Karhulahti, 2012). This can be achieved through immersive storytelling, visuals and audio, and minimal technical glitches. Another important factor is narrative immersion and involvement, where users feel emotionally invested in the virtual world's characters, story, or situations. This emotional connection can be fostered through relatable characters, meaningful interactions, and engaging narratives (Rollings and Adams, 2003; Ryan, 2009). A user's prior VR experience can also affect their perception of realism and believability, with those with more experience having higher expectations for these qualities. Additionally, individuals with vivid imaginations and susceptibility to suggestion are more accepting of realistic and fictional VR experiences (Gilbert, 2016).

Further, several research lines can be pursued to examine the validity and refine the proposed model. Conducting user studies to empirically validate the assumptions about how the proposed quality aspects (plausibility, believability, coherence, congruence) contribute to perceived authenticity and overall quality judgments in VR experiences. Developing standardized scales and questionnaires to quantify and measure the different quality components outlined in the authenticity model (such as perceived realism, logical consistency, emotional resonance, and suspension of disbelief) would also be valuable. Designing controlled experiments systematically manipulating specific variables (e.g., physics accuracy, narrative logic, sensory alignments) to measure their impact on users' perceptions of plausibility, believability, and overall authenticity. Employing multimodal data collection combining subjective reports, behavioral tracking, physiological sensing, and qualitative interviews can capture the multidimensional nature of authenticity assessments. Finally, cross-domain evaluations can help assess the model's applicability and identify potential domain-specific nuances across various VR application areas like training, gaming, therapy, social VR, etc.

## 4 Conclusion

This work puts forth several key findings and contributions. It proposes authenticity as a complementary dimension to presence in evaluating the quality of VR experiences. It argues that while presence focuses on the sense of “being there,” authenticity captures the sense of “trueness and genuineness” felt in the virtual place. It distinguishes between plausibility (adhering to real-world principles, reflecting trueness) and believability (resonating with user perceptions/emotions, capturing genuineness) as two key aspects of authenticity. The paper introduces a structured hierarchy that defines plausibility and believability as subsets under the broader umbrella of authenticity. It also positions coherence and congruence as complementary quality functions related to internal logic (believability) and external mapping (plausibility) respectively. Furthermore, it highlights the importance of considering perceived quality (immediate impressions) and judged quality (reflective evaluations) when assessing authenticity in VR experiences. The work provides a theoretical grounding for holistically evaluating authenticity by spanning perceptual (e.g., graphics, physics), cognitive (e.g., logical consistency, narrative), and emotional (e.g., engagement, resonance) factors.

The present contribution proposes a theoretical model rather than providing empirical validation. While empirical testing is outside the scope of this work, the quality framework for authenticity presented here can inform future research into assessments of VR experience. Comparing the authenticity model against existing frameworks for presence, immersion, realism, etc., to delineate conceptual boundaries and explore potential integrations would be insightful. Also, there is good potential for using empirical findings to iteratively refine and expand the proposed model's theoretical foundations. This paper puts forth a preliminary model to spur additional research that can advance knowledge on factors shaping authentic, high-quality VR user experiences.

## Data availability statement

The original contributions presented in the study are included in the article/supplementary material, further inquiries can be directed to the corresponding author.

## Author contributions

AH: Writing – review & editing, Writing – original draft, Conceptualization, Methodology. AP: Writing – review & editing, Project administration.
